# Viscoelastic Point-of-Care Testing (ClotPro^®^) to Guide Intravenous Thrombolysis in Acute Ischemic Stroke Patients on DOACs: Replacing History with Hemostasis in a Proof-of-Concept Study

**DOI:** 10.3390/neurolint17070103

**Published:** 2025-07-01

**Authors:** Jessica Seetge, Balázs Cséke, Zsófia Nozomi Karádi, Edit Bosnyák, Eszter Johanna Jozifek, László Szapáry

**Affiliations:** 1 Stroke Unit, Department of Neurology, University of Pécs, Ifjúság útja 13, Pécs, 7624 Baranya, Hungary; karadi.zsofia@pte.hu (Z.N.K.); bosnyak.edit@pte.hu (E.B.); jozifek.eszter@pte.hu (E.J.J.); 2 Department of Emergency Medicine, University of Pécs, Ifjúság útja 13, Pécs, 7624 Baranya, Hungary; cseke.balazs@pte.hu

**Keywords:** acute ischemic stroke, intravenous thrombolysis, DOACs, point-of-care testing, ClotPro^®^, propensity score matching, bootstrapping, NIHSS-shift, mRS-shift

## Abstract

Background: Administering intravenous thrombolysis (IVT) in patients with acute ischemic stroke (AIS) on direct oral anticoagulants (DOACs) remains a clinical challenge. Current guidelines restrict IVT within 48 h of DOAC intake unless anticoagulant activity can be confidently excluded. However, reliable medication histories are often unavailable, and conventional coagulation tests inadequately detect DOAC activity. This study evaluated whether viscoelastic point-of-care testing (ClotPro^®^) could identify the absence of anticoagulant effect in AIS patients on DOACs, thus enabling IVT administration and potentially improving clinical outcomes. Methods: We conducted a prospective observational cohort study of 40 AIS patients with documented DOAC use, admitted between February 2023 and May 2025. ClotPro^®^ was performed at admission using the Russell’s viper venom (RVV) assay for factor Xa inhibitors and the ecarin clotting time (ECT) assay for dabigatran. Subtherapeutic anticoagulation was defined as a clotting time (CT) of <100 s for RVV and <180 s for ECT, respectively. Patients identified as being subtherapeutic were assessed for IVT eligibility. To evaluate IVT effects, we performed propensity score-matched bootstrap resampling (1000 iterations), matching patients by age, admission National Institutes of Health Stroke Scale (NIHSS), and pre-stroke modified Rankin Scale (mRS). Primary endpoints were NIHSS-shift (change from admission to 72 h) and mRS-shift (change from pre-stroke mRS to 90-day mRS). Predictors of outcomes were analyzed using multivariate regression models. Results: ClotPro^®^ identified 15/40 patients (37.5%) as subtherapeutic, all on factor Xa inhibitors. Of these, seven received IVT. In matched analyses, IVT-treated patients showed a numerically greater neurological improvement than untreated patients (mean NIHSS-shift: −2.83 vs. 3.94; mean difference: −6.76, 95% confidence interval [CI]: −24.00 to 7.55; *p* = 0.495). Functional outcome by mRS-shift showed only minor differences between groups (2.74 vs. 2.10 mean difference: 0.64; 95% CI: −2.00 to 2.50; *p* = 0.510). IVT showed a favorable trend for early neurological recovery (*p* = 0.081) but was not independently associated with functional outcome (*p* = 0.380). Conclusions: ClotPro^®^ identified a substantial subset of AIS patients on DOAC therapy without measurable anticoagulant activity, enabling IVT in cases that would otherwise have been excluded based on medication history. These findings support the feasibility of ClotPro^®^-guided decision-making in acute stroke care and highlight its potential to improve IVT selection by enabling real-time assessment of coagulation status at the bedside.

## 1. Introduction

Timely reperfusion therapy remains the cornerstone of acute ischemic stroke (AIS) management, with intravenous thrombolysis (IVT) consistently shown to improve clinical outcomes when administered without delay [1] However, in patients receiving direct oral anticoagulants (DOACs), the decision to proceed with IVT is clinically challenging, primarily due to uncertainty regarding anticoagulation status at presentation. Current guidelines recommend withholding IVT within 48 h of last DOAC intake unless anticoagulant activity can be reasonably excluded or a specific reversal agent (e.g., idarucizumab or andexanet alfa) is administered [2]. In real-world practice, both approaches are rarely available in the hyperacute setting, placing clinicians in a time-critical therapeutic dilemma.

This dilemma is further complicated by the nature of stroke presentations. Many patients present with aphasia [3], cognitive impairment [4], or altered mental status [5], making it difficult or impossible to obtain a reliable medication history. Even when patient-reported intake is available, the timing and adherence of the most recent DOAC dose often remain unclear [6].

Standard coagulation assays, including prothrombin time (PT), its derived measure the international normalized ratio (INR), and activated partial thromboplastin time (aPTT), are inadequate for detecting clinically relevant DOAC activity [7,8,9,10]. More precise assessment requires DOAC-specific laboratory assays. For dabigatran, dilute thrombin time [11] and ecarin-based clotting assays [12] are considered most reliable; for factor Xa inhibitors, calibrated anti-Xa assays are the gold standard [13,14,15]. Although liquid chromatography–tandem mass spectrometry (LC-MS/MS) provides highly accurate quantification [16], its limited availability and slow turnaround time make it impractical for urgent decision-making in stroke care [17].

The growing use of DOACs [18], driven by an aging population and rising prevalence of atrial fibrillation [19], has made this diagnostic uncertainty a significant barrier to timely reperfusion therapy. A substantial proportion of AIS patients may be functionally non-anticoagulated at the time of stroke onset due to missed doses, altered pharmacokinetics, or delayed absorption, yet may still be excluded from IVT based solely on reported DOAC use. This cautious, history-based exclusion likely denies many patients potentially beneficial treatment, introduces misclassification bias into clinical research, and may distort the perceived safety and efficacy profile of IVT in this population.

In this context, point-of-care testing (POCT) has emerged as a promising strategy for the rapid bedside evaluation of coagulation status. Viscoelastic POCT platforms, including rotational thromboelastometry (ROTEM^®^) [20], thromboelastography (TEG^®^) [21], and more recently, ClotPro^®^, offer whole-blood assays capable of detecting functional coagulation abnormalities in real time. ClotPro^®^ provides standardized, drug-specific tests: the Russell’s viper venom (RVV) assay for factor Xa inhibitors (e.g., apixaban, rivaroxaban, edoxaban) and the ecarin clotting time (ECT) assay for thrombin inhibitors, such as dabigatran. These assays deliver actionable results within minutes, making them well suited for time-sensitive IVT decisions.

This study aimed to evaluate the feasibility and clinical relevance of POCT in AIS patients with documented DOAC use. Specifically, we investigated whether ClotPro^®^ could reliably detect absent anticoagulant activity in a subset of patients who might be eligible for IVT. Furthermore, we assessed whether IVT was associated with improved neurological or functional outcomes in this real-world cohort. To our knowledge, this represents one of the first investigations into ClotPro^®^-guided IVT decision-making in DOAC-treated AIS patients, a topic of increasing clinical relevance amid the widespread adoption of DOACs and the growing shift toward personalized stroke care.

## 2. Methods

### 2.1. Study Design and Population

This retrospective observational study used data from the Transzlációs Idegtudományi Nemzeti Laboratórium (TINL) STROKE registry at the University of Pécs. Between February 2023 and May 2025, 147 adult patients with AIS and documented use of a DOAC, including apixaban, rivaroxaban, edoxaban, or dabigatran, were registered. Thirteen patients without available 90-day mRS outcome data were excluded, leaving 134 patients eligible for analysis. Of these, 40 consecutive patients underwent POCT (ClotPro^®^) as part of routine clinical care, reflecting the selective application of POCT in time-sensitive stroke workflows. These 40 patients comprised the study cohort.

This study was designed as a proof-of-concept, exploratory analysis to evaluate feasibility, safety, and potential clinical utility of ClotPro^®^-guided IVT decision-making. Accordingly, no formal sample size calculation was conducted, and results should be interpreted in a hypothesis-generating context.

### 2.2. Viscoelastic Point-of-Care Testing with ClotPro^®^

Upon hospital admission, all patients underwent viscoelastic point-of-care testing using the ClotPro^®^ platform (Haemonetics Corporation, Boston, MA, USA; formerly enicor GmbH, Munich, Germany). Blood samples for ClotPro^®^ analysis were collected via venipuncture as citrated whole blood. Simultaneously, INR and glucose levels were assessed at the bedside in the Emergency Department using POCT devices. INR was used as a standard coagulation parameter and occasionally guided the selection of patients for further ClotPro^®^ testing. Glucose measurement offered rapid insight into patients’ glycemic status and was analyzed to explore potential associations with clinical outcomes. These two parameters were specifically highlighted in our analysis due to their clinical relevance.

The ClotPro^®^ analyzer uses pipettes prefilled with starting reagents and 340 μL of citrated whole blood to initiate measurement. The system measures coagulation based on a stationary pin immersed in a rotating cup; reductions in pin movement are detected and plotted as amplitude, generating thrombelastometry curves that reflect real-time coagulation profiles. Clotting time (CT) results for the RVV and ECT assays are typically available within 3 to 5 min after sample insertion, including preparation time, allowing integration into acute stroke workflows without significant treatment delays. Citrated whole blood was used for all assays to ensure sample consistency and reliability.

For patients receiving factor Xa inhibitors, the RVV assay was performed, with subtherapeutic anticoagulation defined as a clotting time of less than 100 s, based on manufacturer guidance [22]. For patients taking dabigatran, the ECT assay would have been used with a threshold of CT < 180 s; however, no such cases were observed during the study period.

### 2.3. Intravenous Thrombolysis Decision Protocol

Patients classified as subtherapeutic based on ClotPro^®^ results were further evaluated for IVT eligibility using established clinical criteria, including time from symptom onset, neuroimaging findings, and the absence of contraindications. Final treatment decisions were made by the attending stroke neurologist in conjunction with the multidisciplinary stroke team. When IVT was administered, alteplase was dosed at 0.9 mg/kg (maximum 90 mg), with 10% delivered as an initial bolus over one minute and the remainder infused over 60 min, following current international guideline recommendations.

### 2.4. Data Collection

Clinical data were collected prospectively, including age, sex, and stroke severity, assessed using the National Institutes of Health Stroke Scale (NIHSS) at admission and at 72 h post-stroke. Functional status was evaluated using the modified Rankin Scale (mRS) at admission (as a proxy for pre-stroke function) and at 90 days post-stroke. Time metrics included onset-to-door, door-to-needle, and door-to-puncture intervals.

Neuroimaging was evaluated using non-contrast computed tomography, with ischemic burden assessed via the modified Alberta Stroke Program Early CT Score (mASPECTS), and collateral circulation graded using the modified Multiphase CT Angiography (mCTA) score.

Admission laboratory data included plasma glucose and INR. Comorbidities such as hypertension, diabetes mellitus, atrial fibrillation, and prior stroke were recorded, along with vascular risk factors, including current smoking and alcohol use. Stroke etiology was classified according to the Trial of ORG 10172 in Acute Stroke Treatment (TOAST) criteria. The specific DOAC used (apixaban, rivaroxaban, edoxaban, or dabigatran) was documented for each patient.

Final acute treatment strategy was categorized as IVT, mechanical thrombectomy (MT), combined therapy (IVT + MT), or standard care (SC) without reperfusion.

### 2.5. Outcome Measures

The primary outcomes were early neurological improvement and long-term functional recovery. Early neurological improvement was defined as the change in NIHSS score from admission to 72 h post-stroke (NIHSS-shift), calculated as 72 h NIHSS minus admission NIHSS, such that more negative values indicated greater improvement. Long-term functional outcome was defined as the change in mRS score from pre-stroke baseline to 90 days (mRS-shift), calculated as 90-day mRS minus pre-stroke mRS, where higher values reflected greater functional decline.

### 2.6. Statistical Analysis

Patients were categorized into subtherapeutic and non-subtherapeutic groups based on ClotPro^®^ thresholds. Continuous variables were reported as means with standard deviations (SD) or medians with interquartile ranges (IQR), depending on distribution assessed via the Shapiro–Wilk test. Categorical variables were expressed as frequencies and percentages. Between-group comparisons were performed using the Student’s *t*-test or Mann–Whitney U test for continuous variables and Fisher’s exact test or chi-squared test for categorical variables. Two-sided *p*-values < 0.05 were considered statistically significant.

To evaluate the effect of IVT among subtherapeutic patients, we performed propensity score matching with bootstrap resampling. Propensity scores were estimated using logistic regression with age, baseline NIHSS, and pre-stroke mRS as predictors. Matching was conducted 1:1 using Mahalanobis distance within a caliper of 0.2 SDs of the logit of the propensity score and stratified by MT status. We performed 1000 bootstrap iterations, and in each matched sample, the effect of IVT on NIHSS-shift and mRS-shift was estimated using linear regression models adjusted for clinically relevant covariates. From these models, we derived average treatment effects, 95% confidence intervals (CIs), and bootstrap-based *p*-values. Covariate balance after matching was assessed using standardized mean differences (SMD), with values < 0.2 considered acceptable.

In addition to matched analyses, multivariable linear regression was performed to identify independent predictors of NIHSS-shift and mRS-shift. Prior to model interpretation, all assumptions of linear regression, including linearity, homoscedasticity, independence of errors, and absence of multicollinearity, were evaluated and confirmed.

All statistical analyses were conducted using Python, version 3.13.

### 2.7. Ethics Approval

This study protocol was approved by the Scientific and Research Ethics Committee of the Medical Research Council of the University of Pécs (approval number: RRF-2.3.1-21-2022-00011, approved on 1 September 2022) and the National Scientific and Research Ethics Committee of Hungary (approval number: BM/22444-1/2024, approved on 1 September 2024). Given the observational design and use of routinely collected clinical data, the requirement for informed consent was waived in accordance with institutional and national ethics regulations.

All treatment decisions, including the administration of IVT in patients with recent DOAC use, were made in routine clinical care according to standardized local protocols. In line with current European Stroke Organisation (ESO) guidelines, IVT was performed only after testing confirmed the absence of clinically relevant anticoagulant activity. In our center, this assessment was supported by point-of-care viscoelastic testing (ClotPro^®^) with drug-specific assays. Thus, all IVT decisions were guideline-compliant and based on individualized safety evaluation.

## 3. Results

### 3.1. ClotPro^®^ Findings and Patient Characteristics

ClotPro^®^ testing identified 15 patients (37.5%) with no detectable anticoagulant activity at admission, all of whom had been receiving factor Xa inhibitors. Within the subgroup without detectable anticoagulant activity, seven patients received IVT, while eight did not. Baseline characteristics for these two groups are presented in Table 1.

Note: In many cases, exact CT values were not documented once clinical thresholds indicating anticoagulant activity were reached, as this reflected routine practice and was sufficient for decision-making. Further details are provided in Section 4.

There were no statistically significant differences between the IVT and no-IVT groups regarding median age (78.0 years [IQR: 75.5–82.0] vs. 81.0 years [IQR: 78.8–84.2]; *p* = 0.449) or sex distribution (male 71% vs. 50%; *p* = 0.608). Stroke severity at admission, measured by median NIHSS scores, was similar between groups (8 [IQR: 4–18] vs. 9 [IQR: 3–10]; *p* = 0.770). Likewise, pre-stroke functional status did not differ significantly (median mRS: 0 [IQR: 0–1] vs. 2 [IQR: 0–3]; *p* = 0.322).

Time-to-treatment metrics showed a significant difference: median onset-to-door time was shorter in the IVT group (90.0 min [IQR: 46.0–116.5]), compared to the no-IVT group (212.0 min [IQR: 136.5–578.5]; *p* = 0.037). The door-to-needle time in the IVT group was 30.0 min (IQR: 26.5–46.0), whereas door-to-puncture times did not differ significantly between groups.

Imaging characteristics were comparable, with a median mASPECTS of 10 in both groups. Excellent collateral circulation (mCTA score 4–5) was observed in 71% of IVT patients and 100% of no-IVT patients (*p* = 0.200).

Admission laboratory values, including plasma glucose (6.50 vs. 7.27 mmol/L; *p* = 0.613) and INR (1.18 vs. 1.15; *p* = 0.862), showed no significant differences. Hypertension and atrial fibrillation were common in both groups. Diabetes mellitus and alcohol use were more frequent in the no-IVT group (75% vs. 29%, *p* = 0.132; and 62% vs. 14%, *p* = 0.119, respectively), but these differences did not reach statistical significance.

Cardioembolic and large-artery atherosclerosis were the predominant stroke etiologies in both groups, with no significant differences in their distribution. All patients were on factor Xa inhibitors (apixaban, edoxaban, or rivaroxaban); none received dabigatran. Regarding treatment allocation, 43% of the IVT group received IVT alone, while 57% underwent combined IVT and MT. In contrast, 38% of the no-IVT group received MT, and 62% were managed with SC.

### 3.2. Propensity Score Matching and Covariate Balance

Of the 1000 bootstrap iterations conducted for propensity score matching, 788 yielded valid matched samples. Covariate balance post-matching was excellent, as reflected by a median SMD of 0.132 and a maximum SMD of 0.187. All covariates met the predefined criterion for acceptable balance (SMD < 0.2), suggesting successful adjustment for confounding.

### 3.3. Clinical Outcomes After Matching

NIHSS scores at 72 h and mRS scores at 90 days were used to compute early neurological and functional outcomes, respectively. Median NIHSS at 72 h was 10 (IQR: 0–14) in the IVT group and 1 (IQR: 0–10) in the no-IVT group (*p* = 0.793). The corresponding 90-day mRS values were 5 (IQR: 4–6) and 2 (IQR: 1–4), respectively (*p* = 0.309). These measures formed the basis of the subsequent NIHSS-shift and mRS-shift analyses.

#### 3.3.1. Neurological Outcome (NIHSS-Shift)

Following matching, the IVT group demonstrated a trend toward greater early neurological improvement. The mean NIHSS-shift was −2.83 in the IVT group, compared to +3.94 in the no-IVT group. The estimated mean difference was −6.76 (95% CI: −24.00 to 7.55; *p* = 0.495). Although this result did not reach statistical significance, the directionality of effect consistently favored IVT across bootstrap samples, suggesting a potential benefit in early neurological recovery.

#### 3.3.2. Functional Outcome (mRS-Shift)

The mean mRS-shift from pre-stroke baseline to 90 days was 2.74 in the IVT group and 2.10 in the no-IVT group (mean difference: 0.64; 95% CI: −2.00 to 2.50; *p* = 0.510). Although this difference was not statistically significant, the observed trend toward worse long-term functional outcomes in the IVT group may be partly attributable to higher mortality within this subgroup. Specifically, among patients with subtherapeutic anticoagulation, 2 of 7 who received IVT died within 90 days, compared to 1 of 8 patients who did not. All deceased patients had also undergone MT, which may indicate greater stroke severity or increased procedural risk. These factors likely contributed to the functional outcomes observed rather than reflecting a direct effect of IVT. Notably, one intracranial hemorrhage occurred in the IVT-treated subgroup, classified radiologically as hemorrhagic infarction type 2 (HI2). However, this event did not meet criteria for symptomatic intracerebral hemorrhage (sICH) as defined by the Heidelberg Bleeding Classification and ECASS III, since it was not associated with clinical deterioration (i.e., no NIHSS increase ≥4 points).

### 3.4. Multivariable Regression Analysis

#### 3.4.1. Predictors of NIHSS-Shift

In the multivariable linear regression model, IVT was associated with a favorable NIHSS-shift, showing a trend toward significance (β = −17.53, *p* = 0.081), consistent with the matched analysis. Older age was associated with less early neurological improvement (β = +1.50, *p* = 0.052). Although none of the covariates reached conventional levels of statistical significance, the model explained a substantial portion of the variance (R^2^ = 0.865; adjusted R^2^ = 0.594), suggesting that it captured meaningful clinical trends. Detailed regression coefficients and confidence intervals are reported in Table 2.

#### 3.4.2. Predictors of mRS-Shift

In contrast, the model predicting mRS-shift did not identify any statistically significant predictors. IVT was not independently associated with long-term functional improvement (β = +2.04, *p* = 0.380), and other variables, including age, admission and 72 h NIHSS, pre-stroke mRS, and onset-to-door time, were also nonsignificant. Despite the lack of statistically significant associations, the model demonstrated strong explanatory power (R^2^ = 0.945; adjusted R^2^ = 0.754), indicating that it explained a substantial portion of the variability in outcome, although the precision of individual effect estimates was limited, likely due to the small sample size. Results of the multivariable model for mRS-shift are presented in Table 3.

## 4. Discussion

### 4.1. Interpretation of Findings

This proof-of-concept study demonstrated the feasibility and potential clinical utility of viscoelastic POCT to guide IVT decisions in AIS patients with documented DOAC use. Among 40 consecutively enrolled patients, ClotPro^®^ identified 15 individuals (37.5%) without detectable anticoagulant activity at presentation, all of whom were on factor Xa inhibitors. Of these, seven patients (47%) were treated with IVT.

Within this subcohort, IVT was associated with greater early neurological improvement, as reflected by more favorable NIHSS-shifts, compared to patients who did not receive thrombolysis. While this trend did not reach statistical significance, it remained consistent across both propensity score-matched and multivariable regression analyses. In contrast, IVT was not associated with improved long-term functional outcomes at 90 days and did not independently predict mRS-shift in adjusted models.

These findings suggest that IVT may offer early neurological benefit in selected patients without measurable anticoagulant activity, even if longer-term functional recovery remains unchanged. While this directional trend is encouraging, particularly for early neurological improvement, it must be interpreted with caution, given the wide confidence intervals and limited statistical power inherent to the small sample size.

### 4.2. Clinical Implications

These results reflect a broader, ongoing challenge in acute stroke management: the frequent disconnect between a patient’s reported DOAC use and their actual anticoagulation status at the time of presentation. In the hyperacute phase of AIS, decisions regarding IVT often must be made without timely or reliable information on anticoagulant activity. As a result, patients who are functionally non-anticoagulated may be inappropriately excluded from potentially beneficial therapy based solely on medication history.

ClotPro^®^ offers a practical, drug-specific solution to this diagnostic uncertainty. With rapid turnaround times and dedicated assays, RVV for factor Xa inhibitors and ECT for direct thrombin inhibitors, it enables real-time bedside assessment of anticoagulation. Importantly, the aim is not to restrict IVT access but to expand it by identifying patients who may safely receive treatment despite incomplete or outdated histories. This represents a shift from a rigid, history-based exclusion paradigm to a more precise, physiology-driven inclusion framework.

Recent retrospective studies have challenged the traditional caution surrounding IVT in DOAC-treated patients, suggesting that thrombolysis may be safe, even within 48 h of DOAC intake [23,24,25]. These findings are encouraging and indicate that the risks of treatment may be lower than previously assumed. However, most of these studies have involved highly selected cohorts, typically patients with low or borderline plasma DOAC levels or only indirect evidence of anticoagulant activity (often inferred from history rather than direct measurement). As a result, their findings cannot be generalized to the broader DOAC-treated population, particularly patients with plasma levels >100 ng/mL, who remain largely underrepresented due to bleeding risk concerns. Until large randomized controlled trials (RCTs) definitively establish the safety of IVT across the full spectrum of anticoagulation, adopting a universal “treat regardless” approach remains speculative and potentially unsafe.

In this context, our study provides a pragmatic middle ground. By using ClotPro^®^ to identify patients who are functionally non-anticoagulated, IVT can be administered more confidently and more often, without unnecessary delays or blanket exclusions. Rather than contradicting broader observational trends, this strategy complements them. It supports a precision-based approach to treatment expansion, grounded in individualized assessment rather than assumptions.

Ultimately, this approach reflects the direction of modern stroke care: timely, tailored interventions guided by real-time patient physiology. ClotPro^®^ represents a feasible and scalable tool for navigating the current evidence gap, bridging the limitations of retrospective data and the promise of future RCTs while enabling safe, individualized decision-making in the present.

### 4.3. Comparison with Existing Literature

To place these findings in context, it is important to consider the growing body of literature on viscoelastic testing and POCT tools for DOAC management in AIS.

Previous studies have highlighted the limitations of relying solely on patient-reported medication history or standard coagulation assays, both of which may fail to accurately reflect anticoagulant status during acute stroke evaluation. Viscoelastic POCT platforms such as ClotPro^®^ were developed to address these limitations.

A systematic review by Sahli et al. evaluated 53 studies on the influence of DOACs on POCT and emphasized the need for drug-specific assays. Notably, the ClotPro^®^ RVV test for factor Xa inhibitors and the ECT test for thrombin inhibitors were identified as providing clinically meaningful bedside data with implications for acute therapeutic decision-making [26].

Heubner et al. extended this work by demonstrating that POCT can accurately detect residual anti-Xa activity following DOAC reversal, supporting its use in time-sensitive contexts such as thrombolysis [27].

In AIS specifically, Sedghi et al. proposed a prospective study to assess whether ClotPro^®^ could reliably identify patients without significant anticoagulant effects in the hyperacute setting [28]. This reflects a broader clinical shift away from fixed time windows and toward real-time, physiology-based assessment.

Other POCT methods have also shown promise. A recent pilot study assessed a urine-based DOAC dipstick in AIS patients and reported high sensitivity and specificity for detecting anticoagulant activity [29]. Notably, the test reduced time to treatment decisions by more than two hours compared to blood-based assays, underscoring the practical advantages of rapid bedside diagnostics.

### 4.4. Limitations

This study has several limitations that must be acknowledged. First, its retrospective, observational design precludes any causal inference regarding the effect of IVT. Although we attempted to emulate a target trial framework using propensity score matching, Mahalanobis distance, and bootstrap resampling, the possibility of residual confounding from unmeasured variables cannot be excluded. Furthermore, clinical decisions to administer or withhold IVT may have been influenced by factors not captured in our dataset.

Second, anticoagulation status was assessed using the viscoelastic POCT platform ClotPro^®^ rather than LC-MS/MS, the pharmacokinetic gold standard for DOAC quantification. While ClotPro^®^ assays are validated and provide functionally relevant data, they may fail to detect low-level anticoagulant activity measurable by more sensitive laboratory methods.

Third, the relatively small sample size, particularly within the subtherapeutic IVT-treated subgroup, limited statistical power to detect modest treatment effects. The absence of patients treated with dabigatran further narrows the scope of inference to factor Xa inhibitors, and the single-center setting may constrain the generalizability of our findings. As a result, all findings should be interpreted as preliminary and hypothesis-generating, with directional trends warranting validation in larger, prospective studies.

Fourth, while exact ClotPro^®^ CT values were not consistently documented for all patients, this reflects routine clinical practice rather than a study limitation. Exact CTs were available in 11 of 15 patients with subtherapeutic anticoagulation and in 7 of 25 patients with detectable anticoagulant activity; in other cases, threshold-based values (e.g., “<100 s” or “>100 s”) were recorded. Urgent treatment decisions in acute stroke care are commonly guided by these established CT thresholds, making precise values beyond these cutoffs clinically unnecessary. Furthermore, ClotPro^®^ results are stored only temporarily and not automatically archived, limiting retrospective retrieval of undocumented values. Importantly, this approach did not compromise anticoagulation classification or treatment decisions and reflects the real-world use of viscoelastic POCT in emergency settings.

Despite these limitations, the internal validity of our findings is supported by strong covariate balance after matching and the consistency of results across descriptive, matched, and multivariable analyses. Together, these strengths provide a robust foundation for future prospective, multicenter studies to validate the clinical utility of POCT-guided anticoagulation assessment in acute stroke care.

## 5. Conclusions

Viscoelastic POCT provides a rapid, bedside method for the individualized assessment of anticoagulant activity in AIS patients with reported DOAC use. In this study, it identified a meaningful subgroup without measurable anticoagulant effect, patients who may otherwise have been excluded from thrombolysis. Among these individuals, IVT was associated with improved early neurological outcomes. These findings support the clinical use of ClotPro^®^ in acute stroke care and show its potential to guide IVT decisions based on actual coagulation status rather than medication history alone.

## Figures and Tables

**Table 1:** Baseline characteristics of subtherapeutic patients by IVT status.

Variable	IVT Group (*n* = 7)	No-IVT Group (*n* = 8)	*p*-Value
Demographics			
Age, years, median (IQR)	78.0 (75.5–82.0)	81.0 (78.8–84.2)	0.449
Sex, male, *n* (%)	5 (71%)	4 (50%)	0.608
Stroke Severity and Function, median (IQR)			
NIHSS at admission	8 (4–18)	9 (3–10)	0.770
Pre-stroke mRS	0 (0–1)	2 (0–3)	0.322
Time Metrics, min, median (IQR)			
Onset-to-door time	90.0 (46.0–116.5)	212.0 (136.5–578.5)	0.037 *
Door-to-needle time	30.0 (26.5–46.0)	-	-
Door-to-puncture time	110.0 (98.0–123.5)	110.0 (104.5–183.5)	0.857
Neuroimaging Findings, *n* (%)			
mASPECTS	10 (8–10)	10 (8–10)	0.999
10	4 (57%)	5 (62%)	0.999
9	0 (0%)	0 (0%)	0.999
8	2 (29%)	1 (12%)	0.569
7	0 (0%)	1 (12%)	0.999
6	0 (0%)	0 (0%)	0.999
5	1 (14%)	1 (12%)	0.999
mCTA score			
4–5	5 (71%)	8 (100%)	0.200
2–3	2 (29%)	0 (0%)	0.200
0–1	0 (0%)	0 (0%)	0.999
Admission Laboratory Values, median (IQR)			
Plasma glucose, mmol/L	6.50 (6.18–7.30)	7.27 (6.28–8.11)	0.613
INR	1.18 (0.96–1.23)	1.15 (1.05–1.20)	0.862
Medical History, *n* (%)			
Hypertension	7 (100%)	8 (100%)	0.999
Diabetes mellitus	2 (29%)	6 (75%)	0.132
Atrial fibrillation	6 (86%)	8 (100%)	0.467
Previous stroke	1 (14%)	2 (25%)	0.999
Current smoking	0 (0%)	0 (0%)	0.999
Alcohol use	1 (14%)	5 (62%)	0.119
Stroke Etiology, *n* (%)			
Cardioembolic	6 (86%)	6 (75%)	0.999
Large-artery atherosclerosis	0 (0%)	1 (12%)	0.999
DOAC Type, *n* (%)			
Apixaban	5 (71%)	6 (75%)	0.999
Edoxaban	1 (14%)	1 (12%)	0.999
Rivaroxaban	1 (14%)	1 (12%)	0.999
Dabigatran	0 (0%)	0 (0%)	0.999
Recanalization Therapy, *n* (%)			
IVT	3 (43%)	0 (0%)	0.077
MT	0 (0%)	3 (38%)	0.200
IVT + MT	4 (57%)	0 (0%)	0.026 *
SC	0 (0%)	5 (62%)	0.026 *

* indicates statistical significance (*p* < 0.05). Abbreviations: IVT = intravenous thrombolysis, IQR = interquartile range, NIHSS = National Institutes of Stroke Health Scale, mRS = modified Rankin Scale, mASPECTS = modified Alberta Stroke Program Early CT Score, mCTA = modified Multiphase Computed Tomography Angiography, INR = international normalized ratio, DOAC = direct oral anticoagulant, MT = mechanical thrombectomy, SC = standard care.

**Table 2:** Predictors of NIHSS-shift.

Variable	Coefficient	*p*-Value	95% CI
Age	1.4957	0.052	−0.030 to 3.021
NIHSS at admission	−1.0466	0.081	−2.332 to 0.238
Pre-stroke mRS	−2.6025	0.547	−14.829 to 9.624
Onset-to-door time	0.0082	0.937	−0.299 to 0.316
IVT	−17.5296	0.081	−39.039 to 3.980
MT	−4.0200	0.779	−45.727 to 37.687

Abbreviations: NIHSS = National Institutes of Stroke Health Scale, CI = confidence interval, mRS = modified Rankin Scale, IVT = intravenous thrombolysis, MT = mechanical thrombectomy.

**Table 3:** Predictors of mRS-shift.

Variable	Coefficient	*p*-Value	95% CI
Age	0.1273	0.481	−0.511 to 0.766
NIHSS at admission	0.0598	0.429	−0.202 to 0.321
NIHSS at 72 h	0.1018	0.361	−0.271 to 0.475
Pre-stroke mRS	−0.3725	0.609	−3.040 to 2.295
Onset-to-door time	−0.0006	0.971	−0.063 to 0.062
IVT	2.0412	0.380	−5.827 to 9.910
MT	1.4433	0.545	−7.162 to 10.048

Abbreviations: mRS = modified Rankin Scale, CI = confidence interval, NIHSS = National Institutes of Stroke Health Scale, IVT = intravenous thrombolysis, MT = mechanical thrombectomy.

## Data Availability

The data supporting the findings of this study are provided within the manuscript. For any additional requests regarding raw data, please contact the corresponding authors.

## Abbreviations

IVT	intravenous thrombolysis
AIS	acute ischemic stroke
DOAC	direct oral anticoagulant
RVV	Russell’s viper venom
ECT	ecarin clotting time
CT	clotting time
NIHSS	National Institutes of Health Stroke Scale
mRS	modified Rankin Scale
CI	confidence interval
PT	prothrombin time
INR	international normalized ratio
aPTT	activated partial thromboplastin time
LC-MS/MS	liquid chromatography–mass spectrometry/mass spectrometry
POCT	point-of-care testing
TINL	Transzlációs Idegtudományi Nemzeti Laboratórium
mASPECTS	modified Alberta Stroke Program Early CT Score
mCTA	modified Multiphase CT Angiography
TOAST	Trial of ORG 10172 in Acute Stroke Treatment
MT	mechanical thrombectomy
SC	standard care
SD	standard deviation
IQR	interquartile range
SMD	standardized mean difference
ESO	European Stroke Organisation
HI2	hemorrhagic infarction type 2
sICH	symptomatic intracerebral hemorrhage
RCT	randomized controlled trial
